# Promoting shared decision-making in colorectal cancer screening in primary care: A cluster randomized controlled trial

**DOI:** 10.1371/journal.pone.0351069

**Published:** 2026-06-09

**Authors:** Tamara Scharf, Yonas Martin, Marc-Andrea Janggen, Adrian Rohrbasser, Kali Tal, Nikola Biller-Andorno, Jean-Luc Bulliard, Kevin Selby, Reto Auer

**Affiliations:** 1 Institute of Primary Health Care (BIHAM), University of Bern, Bern, Switzerland; 2 Graduate School of Health Sciences, University of Bern, Bern, Switzerland; 3 Department of Infectious Diseases, Bern University Hospital, University of Bern, Bern, Switzerland; 4 Institute for Biomedical Ethics and History of Medicine (IBME), UZH, Zürich, Switzerland; 5 Center for Primary Care and Public Health (Unisanté), Lausanne, Switzerland; Shuguang Hospital, CHINA

## Abstract

**Introduction:**

In Switzerland, primary care physicians (PCP) prescribe colonoscopy for colorectal cancer (CRC) screening rather than offering a choice between colonoscopy and faecal occult blood test (FOBT). This study evaluated a training program promoting shared decision-making for CRC screening.

**Methods:**

PCP from a research network were randomized 1:1 into intervention or control. The intervention group received study materials, patient decision aids, evidence summary, FOBT sample kit, and personalized feedback on CRC screening practices. PCP documented CRC screening decisions of 40 consecutive patients (ages 50–75) four months post-intervention. The control group received no materials before data collection.

**Results:**

Of 110 PCP randomized, 83 (76%) collected data on 3,171 patients (mean age 62, 50% women). PCP in the intervention group were more likely than controls to have at least one patient tested or planning FOBT (84% vs. 56%; unadjusted RR: 1.52; 95% CI: 1.13 to 2.04). In a sensitivity analysis restricted to 62 PCP who participated in a previous data collection, 72% (21/29) already met the primary outcome in the intervention group at baseline and 49% (16/33) in the control group (RR: 1.49; 95% CI: 0.98 to 2.28). When contrasting the change within PCP from the 2017 and 2018 data collection, there was no significant increase in proportion of PCP who met primary outcome between intervention and control group, while it might have increased the proportion of PCP already prescribing FOBT to prescribe it to more of their patients.

**Conclusion:**

A mailed intervention increased FOBT prescriptions, but selection bias may have influenced results.

## Introduction

Colorectal cancer (CRC) is the third most common cause of cancer death in high-income countries, but absolute risk of death from CRC can be halved if people between the ages of 50 and 80 are screened with either colonoscopy or fecal occult blood test (FOBT), which detects human blood in stool [1]. International Guidelines as well as the Swiss cancer foundation recommend patients be offered the choice between colonoscopy and FOBT. In screening programs colonoscopy and FOBT perform similarly in detecting CRC, but colonoscopy is more likely to detect polyps [2–4]. If both options are available, patients divide about evenly in preference [5,6]. Since both tests can reduce CRC mortality, choice of test should be considered “preference sensitive”—physicians should offer both and let patients decide which test they prefer [7].

Colonoscopy is by far the most common testing method in Switzerland and in large portions of the United States, suggesting that primary care physicians (PCP) rarely offer their patients the opportunity to make an informed choice between methods, and may even advise against FOBT [8,9]. An analysis of Swiss 10-year testing rates showed that the increase in CRC screening rate from 2007 (33.2%) to 2017 (48.4%) was driven by an increase in colonoscopy. At the same time, FOBT testing rates declined [10].

In previous studies focusing on Swiss PCP, we highlighted high variation in testing methods and that prescribing only colonoscopy was associated with higher rates of screening refusal [8,9]. A randomized trial conducted in the US showed that screening uptake increased from 38% (colonoscopy alone) to 69% if patients were offered a choice between colonoscopy and FOBT, mainly driven by an increase in FOBT [11].

We developed an intervention to encourage PCP to give patients a choice between CRC screening tests. As mailed interventions to improve CRC screening offer a simple but efficient outreach approach and remain largely underexplored, we decided to deliver our intervention by mail. The intervention comprised evidence summaries for clinicians, a decision aid that facilitated shared decision-making (SDM) and personalized performance feedback. SDM is a collaborative decision-making process in which patients and healthcare professionals together consider evidence and select treatments that match the patients’ values and preferences. This method encourages clinicians to present the treatment or screening options based on the best available evidence and solicit and integrate patient’s preferences and values about treatment options for healthcare decisions [7,12,13]. If Swiss PCP can be taught to facilitate SDM in discussions of CRC with their patients, more of their patients will be able to choose the test which better reflects their personal preferences and values. If PCP have none of their patients prescribed a FOBT and only have patients screened with colonoscopy, we can reasonably posit that they did not offer the choice of FOBT and that they did not engage in SDM about choice of test. We can use FOBT prescriptions as a way of tracking SDM in CRC screening decisions.

Increasing the number of PCP who offer FOBT could raise Switzerland’s low overall screening rate. Our cluster randomized controlled trial was based on previous work we conducted among PCP in Switzerland [8,9]. We set out to determine if our mailed training intervention would increase the proportion of PCP who prescribed FOBT to their patients and their intention to prescribe FOBT, from which we would infer that they engaged in SDM for CRC screening.

## Methods

### Study design and randomization

This was a randomized controlled clinical trial on CRC screening among PCP of the Swiss wide Sentinel Surveillance Network (Sentinella) with a 1:1 allocation ratio. The Clinical Trials Unit of the University of Bern (Switzerland) used the R software to independently randomize participants at the practice level. This assured that PCP who worked in the same practice would not be randomized to different groups, minimizing risk of co-intervention. We stratified for PCP who worked in a canton with an ongoing or planned organized CRC screening program, and for PCP who had participated in intervention-free data collection in 2017 [8].

### Participants

We invited general practitioners of the Sentinella network to participate in our study, via the Federal Office of Public Health (FOPH). Sentinella is a cooperative practice-based surveillance network of PCP coordinated by the FOPH that is designed to monitor infectious diseases in the population. PCP in Sentinella regularly report data on infections on their patients and participate in further data collection such as the one we proposed. We used the same recruitment methods as in our 2017 study with Sentinella PCP [9]. PCP were eligible if they worked with adults and did not practice in either Uri and Vaud cantons, which already have special cantonal CRC screening programs that promote FOBT. PCP could opt out of the study by mailing a prefilled form of declination to the FOPH. PCP in the Sentinella practice-based research network receive a yearly lump sum for all data collection activities, so there was no additional incentive for their optional participation in data collection on CRC screening and in our trial. Participating PCP reported irreversibly anonymized patient data collected during consultations. The FOPH provided us with data at the PCP level, including PCP sex, age group (30–39, 40–49, 50–59, and 60 and above), language region (German, French, Italian), and area of practice (urban, intermediate, rural). The Federal Statistics Office defines location categories based on size, population density, and accessibility [14].

### Data collection

We asked each physician to systematically include 40 consecutive 50–75-year-old patients seen for a non-urgent face-to-face consultation that lasted more than five minutes. PCP who felt overburdened by including every eligible consecutive patient could instead include the first two patients per half-day of work. As in our early study, we collected data from PCP via a printed paper-based form (see S1 Fig) developed in collaboration with PCP and specialists [8,9]. The form (see S2 Fig) was based on an algorithm that integrated FOBT into PCP’s daily routine and guided them to collect the minimum amount of data we needed. We refined the form during a pilot test with 10 PCP [15]. To familiarize PCP with the form, we gave them examples of completed forms based on short clinical vignettes. The examples were also intended to increase the standardization of data collection throughout the Sentinella network. Following Sentinella’s usual process for data collection, we sent the form to the FOPH and the FOPH distributed it to participating PCP in October 2018, 4 months after the intervention group had received the intervention package (see below). This data collection was the same as the data collection performed in 2017, to which a subsample of randomized PCP participated [8,9]. Physicians sent completed forms to the FOPH, which transferred the forms to our research group to ensure the data was doubly anonymized. Outcome data collection began in October 2018 and ended in January 2019.

PCP reported the number of weeks it took them to collect data from all 40 patients, and reported patient age, sex, and previous CRC testing. If patients had been tested within recommended intervals, PCP collected no more data. If patients had not been previously tested, PCP reported if CRC screening was contra-indicated. If patients were eligible for a discussion about CRC screening, PCP reported the discussion or explained why they did not discuss screening. During a discussion, PCP collected data on presence of potential CRC symptoms and risk factors for CRC. If patients refused screening, PCP noted the reason. Finally, PCP indicated whether patients planned to be screened and their chosen method (see S1 and S2 Fig, further description available in Martin et al. 2019) [15].

PCP reported their prescribing intentions, knowledge about CRC screening options, and use of intervention materials on a separate questionnaire that the FOPH sent with the data collection forms.

### Intervention

PCP in the intervention group received a postal package in June 2018 that contained: our study rationale; a 2-page structured evidence summary with information on CRC screening (colonoscopy and FOBT; see S3 Fig); patient decision aid (20-page booklet) to distribute during the discussion; a plasticized 4-page abridged version of our booklet, designed to support PCP when they discussed CRC screening with patients during a clinical visit (see S4 Fig); a 2-page document that explained why PCP who still used the inferior guaiac-based FOBT (gFOBT) should switch to the immunological FOBT (iFOBT, also called FIT), supplemented by a list of laboratories from which they could order the suggested iFOBT; a sample iFOBT; a video that exemplified a participatory approach to discussing CRC screening with a patient (provided on a USB stick); (only for PCP who participated in our 2017 data collection) an individualized 1-page summary of PCP screening practices that compared their own prescription patterns to group patterns (see S5 Fig). The training material and patient decision aids build on previous developments promoting shared decision making in colorectal cancer screening [16–18].

### Control

The control group received no intervention. PCP in this group were only invited to collect outcome data on consultations with 40 consecutive 50–75-year-old patients. Data collection was done at the same time than in the intervention group.

### Outcomes

Our pre-registered primary outcome was the number of PCP who, during the data collection period, had at least one patient tested with FOBT, or had prescribed at least one FOBT to eligible patients (ClinicalTrials.gov ID: NCT03552744). We thus intended to track changes in FOBT prescription that might have occurred during the 4–6 months between the time the intervention package was mailed to PCP and the time outcome data was collected during the consultation (track FOBT prescribed and performed among patients who might have come for a visit within these 4–6 months and came back for a visit when outcome data was collected AND patients not previously tested to whom PCP prescribed a FOBT during the consultation when outcome data was collected). Secondary outcomes were 1) the number of PCP for whom the proportion of their patients previously tested or planned to be tested was at least 40% at the time of the clinical visit, 2) the number of PCP who discussed CRC with at least 50% of their eligible patients and 3) the proportion of patients where FOBT was planned after discussion. Further secondary outcomes were 4) PCP’s intentions to prescribe to ≥ 40% of their patients, and 5) PCP’ intentions to prescribe colonoscopy vs. FOBT over the next six months. These two last secondary outcomes were collected via the separate questionnaire sent to PCP. Outcome cut-offs were chosen arbitrarily during sample size and power calculation, before the trial began.

### Deviation from the protocol

Although PCP systematically collected data to compute this outcome, we did not plan to include the rate of patients up-to-date for CRC screening (screened for CRC within recommended intervals; FOBT< 2 years or colonoscopy <10 years) and the proportion of patients where FOBT was planned after discussion, as secondary outcomes in the analyses at the time we planned and registered the trial. We did not expect to be powered for this outcome; instead, we added a posteriori in the analyses because it added context to our main results and their interpretation [19].

### Statistical analysis

We used descriptive statistics to characterize the PCP who participated in the trial and the patients they included, and analyzed outcomes based on the intention-to-treat principle. For the primary outcome, we estimated each PCP’ overall prescription of FOBT, based on their reported tests (FOBT vs. colonoscopy) for their 40 patients and the rate of FOBT prescribed after discussion. We dichotomized this covariate into never-prescribed FOBT (none of the 40 patients had been tested with FOBT and the PCP did not prescribe FOBT to any of these patients after discussion) and prescribed FOBT (any of the 40 patients had been tested with FOBT or the PCP prescribed FOBT after discussion). We used Poisson regression with robust error variance to estimate relative risk of the intervention vs. control for outcomes at the PCP level, adjusting for PCP characteristics such as age, gender, area of practice (urban/intermediate/rural), and language region (German/French/Italian). We further adjusted for patient’s age and gender for overall screening rates. All analyses were by originally assigned groups.

### Sample size calculation and Sensitivity Analysis

Given that the number of eligible PCP was fixed by the size of the network, we calculated the sample size we needed to detect significant differences between groups. We assumed about 50% relative reduction in number of PCP’s who only prescribed colonoscopy, from 49% to 26% (23% absolute risk difference). Enrolling at least 86 PCP (43 PCP per group) would give us more than 80% power to detect a difference.

We tested the sensitivity of the results to the inclusion of data from PCP that participated in our 2017 data collection and in the trial. We tested whether the randomization of PCP in 2017 (baseline) was balanced for the primary outcome by computing the proportion of PCP in each randomized group who had at least one patient tested with FOBT or had prescribed at least one FOBT to eligible patients (primary outcome). We then tested whether selective attrition might explain significant differences between groups by restricting these 2017 data analyses on the primary outcome to those who participated in the 2017 and 2018 data collections. We further repeated these analyses for CRC screening within recommended intervals (FOBT< 2 years or colonoscopy <10 years) (unplanned secondary outcome). Lastly, we explored the difference in the proportion of patients who were tested with FOBT or planned to be tested with FOBT between 2017 and 2018 at an aggregate, patient-level, by randomized group (i.e., not considering the clustering by PCP) among PCP who participated in both data collections.

We conducted all statistical analyses with Stata version 15.1 (StataCorp). We considered a 2-sided P < .05 statistically significant and started our analysis in January 2019.

### Ethics approval and consent to participate

The ethics committee of Canton Bern (CH) waived ethical approval for the study because we doubly and irreversibly anonymized patient data (FOPH cannot identify patients and we cannot identify PCP). Our study thus fell outside of the scope of Switzerland’s Human Research Act (Req-2018–00409).

## Results

**Fig 1:**
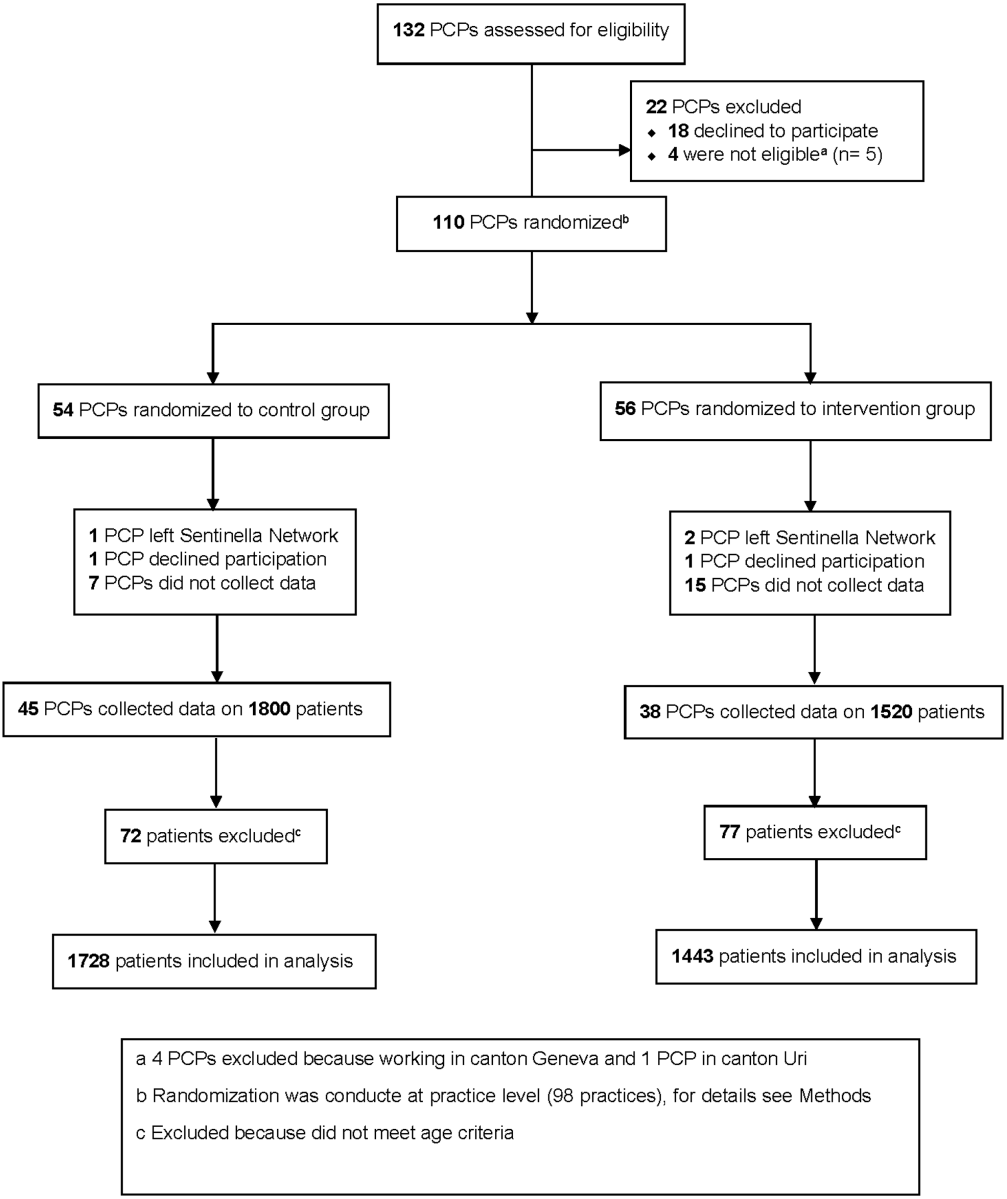
Flowchart. The Flowchart showing enrollment, randomization, exclusion, analysis of participating PCPs and collected patient data in the intervention and control groups.

Of 132 PCP evaluated for eligibility, 128 were asked to decline participation in a mailed form (after excluding 4 PCP who worked in the canton of Vaud and Uri with ongoing CRC screening programs) and 110 did not decline to participate. We randomized 96 PCP practices, 28 PCP worked with other PCP in the same practice (14 PCP practices each with max 2 participating PCP, see Fig 1). Among the 110 PCP included, 74 PCP had participated in the earlier 2017 data collection. After dropouts, 45 PCP (4 PCP in 2 group practices) in the control group collected data on 1800 patients and 38 PCP (6 PCP from 3 group practices) in the intervention group collected data on 1520 patients. Among the 83 PCP who collected data in 2018, 63 (76%) (30 in intervention group and 33 in control group) collected data in the 2017 data collection [9]. We excluded data from 72 patients in the control group and 77 patients in the intervention group because they did not meet age criteria. Baseline characteristics of PCP and patients are reported in Table 1. Mean age of PCP was 54; 25% were women, most PCP (72%) worked in urban areas in the German-speaking regions (65%) of Switzerland. Mean age of patients was 62; patients were divided equally between men and women.

**Table 1:** Baseline characteristics of primary care physicians (PCP) and patients participating in the trial.

PCP characteristics	Control (N = 45)	Intervention (N = 38)
Age, mean*	54.7	53.9
Women – n (%)	10 (22.2)	11 (28.9)
Language region		
German – n (%)	31 (68.9)	23 (60.5)
French – n (%)	9 (20.0)	12 (31.6)
Italian – n (%)	5 (11.1)	3 (7.9)
Area of practice		
Urban – n (%)	32 (71.1)	28 (73.7)
Intermediate – n (%)	6 (13.3)	7 (18.4)
Rural – n (%)	7 (15.6)	3 (7.9)
**Patient characteristics**	**Control (N = 1,728)**	**Intervention (N = 1,443)**
Age, mean	62.2	62.3
Women – n (%)	869 (50.3)	724 (50.1)

N indicates the total number of physicians per randomized group. n indicates the number of physicians within the specified subgroup.

*Mean age of PCPs was estimated from grouped age ranges using midpoints of each age group; results should be interpreted as approximate.

### Primary outcome

**Fig 2:**
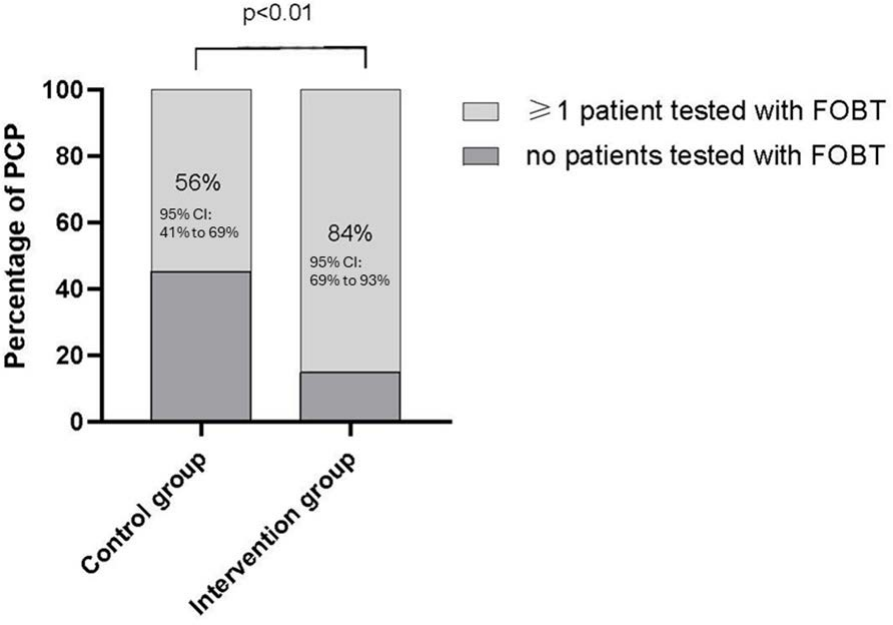
Primary Outcome: Proportion of PCP who had at least one patient previously tested with FOBT, or who prescribed at least one FOBT to eligible patients during the data collection period. Results stratified by randomized groups.

**Fig 3:**
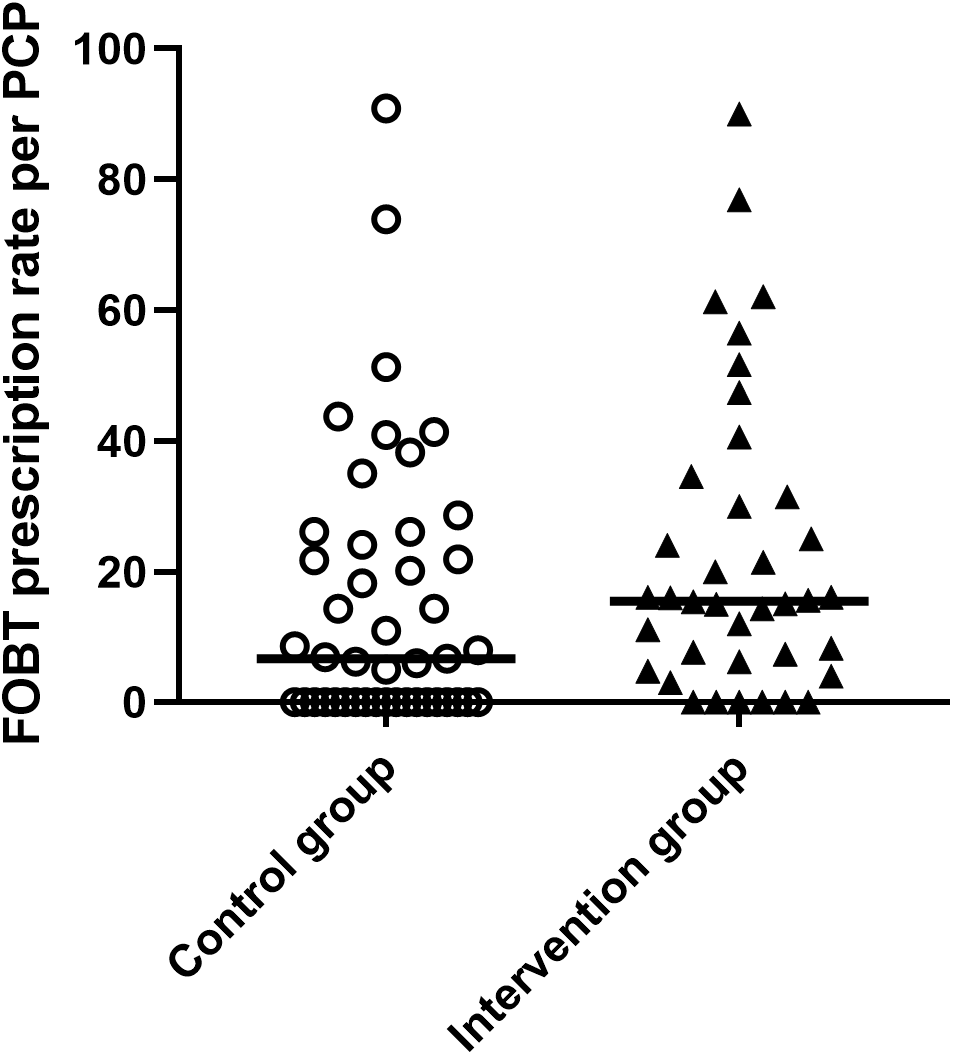
Distribution PCP who had at least one patient previously tested with FOBT, or who prescribed at least one FOBT to eligible patients during the data collection period. Results stratified by randomized groups. Each point represents one PCP and the proportion of eligible patients with previous or newly prescribed FOBT during the data collection period.

In the main pre-defined analyses on 2018 data, PCP in the intervention group were more likely to have at least one patient previously tested with FOBT, or to have prescribed at least one FOBT to eligible patients during the data collection period (32/38; 84%; 95% CI: 69% to 93%), than PCP in the control group (25/45; 56%; 95% CI: 41% to 69%); difference in absolute risk was 29% (unadjusted RR: 1.52; 95% CI: 1.13 to 2.04) (see Figs 2–3 and Table 2).

**Table 2:** CRC screening outcomes.

			Unadjusted		Multivariable adjusted*	
PCP: N = 83	Control (N = 45)	Intervention (N = 38)	Relative risk (95% CI)	p-value	Relative risk (95% CI)	p-value
PCP who had at least one patient previously tested with FOBT, or who prescribed at least one FOBT to eligible patients – n (%)	25 (55.5)	32 (84.2)	1.52 (1.13-2.04)	0.006	1.46 (1.08-1.98)	0.015
PCP whose proportion of patients tested before the clinical visit or planning to be tested during the visit with FOBT vs colonoscopy was at least 40% - n (%)	19 (42.2)	23 (60.5)	1.43 (0.94-2.20)	0.100	1.43 (0.94-2.18)	0.097
PCP intending to prescribe FOBT to >40% of their patients over the next 6 months – n (%)	10 (22.2)	21 (55.3)	2.49 (1.34-4.63)	0.004	2.37 (1.33-4.24)	0.004
Patients up to date with CRC testing before discussion – n/N (%)	829/1728 (48.0)	785/1443 (54.4)	1.13 (1.06-1.21)	<0.001	1.11 (1.04-1.19)**	0.003

N indicates the total number of physicians or patients per randomized group. n indicates the number within the specified subgroup.

* Results from multivariate adjusted Poisson regression models, adjusted for PCP characteristics such as age, gender, area of practice (urban/intermediate/rural), and language region German/French/Italian).

** Models additionally adjusted for age and gender of patients.

Results were similar when restricting the sample to only PCP who participated in both the 2017 and 2018 data collection for the primary outcome in 2018 (24/29; 83% vs 18/33; 55%; RR: 1.52; 95% CI: 1.06 to 2.17, see S3 Table). However, results on data collected in 2017 (before the intervention took place) among PCP randomized (including PCP who did not collect data in 2018) suggest a first possible imbalance in randomization due to chance on the primary outcome: 65% (24/37) of PCP in intervention group and 51% (19/37) in the control group already had met primary outcome in 2017 (RR: 1.26, 95% CI: 0.85 to 1.88, see S3 Table). Also, when restricting to the 62 PCP randomized and who participated in both 2017 and 2018 data collection, the point estimate was similar for the main analyses, suggesting selective attrition of PCP (see S3 Table). In intervention group, 72% (21/29) of PCP met primary outcome in 2017 and 49% (16/33) in the control group (RR: 1.49, 95% CI: 0.98 to 2.28, see S3 Table). Finally, when contrasting the change within PCP from the 2017 and 2018 data, there was no significant increase in proportion of PCP who met primary outcome between intervention and control group (see S3 Table).

### Secondary outcomes

(1) Proportion of PCP with higher than 40% FOBT prescription rates was similar in both groups in 2018 (42.2% vs. 60.5% for the control and intervention group, respectively; unadjusted: RR: 1.43; 95% CI: 0.94 to 2.20). In addition, among PCP who participated in both data collections 2017 and 2018, the proportion of PCP whose patients had ≥ 40% FOBT use increased in the intervention group (from 55.2% to 62.1%) but remained unchanged in the control group (39.4% in both years) (S4 Table). (2) The number of PCP who discussed CRC screening with at least 50% of their eligible patients was similar in both groups (47.4% in the intervention group; 55.6% in the control group; unadjusted: RR: 0.85; 95% CI: 0.56 to 1.31). (3) The proportion of patients where a FOBT was planned after discussion was 32% in the control group and 61% in the intervention group (Table 3). When restricting analyses to PCP who participated in both the 2017 and 2018 data collections, 39.9% (65/163) of patients of PCP in the control group planned FOBT vs 50% (73/146) of patients of PCP randomized into the intervention group in 2017. In 2018, after randomization, the difference between groups decreased to 36% (50/139) of patients of PCP in the control group and increased to 65.6% (63/96) in the intervention group (S5 Table). (4) Overall, the intervention had no statistically significant effect on PCP intention to prescribe CRC screening tests (57.9% vs. 46.7% in the intervention and control group, respectively; unadjusted RR: 1.24; 95% CI: 0.82 to 1.88). (5) PCP in the intervention group were more likely to intend to prescribe FOBT to ≥ 40% of their patients than PCP in the control group (55% intervention vs. 22% control; unadjusted RR: 2.49; 95% CI: 1.34 to 4.63) (see Table 2, adjusted results).

**Table 3:** Details on discussion about CRC screening by trial arm.

	Control	Intervention
**Total patients included – N (%)**	**1728 (100)**	**1443 (100)**
Patients up to date with CRC testing before discussion – n (%)	829 (48.0)	785 (54.4)
- *Colonoscopy – n (%)*	*734 (42.5)*	*680 (47.1)*
- *FOBT/FIT – n (%)*	*95 (5.5)*	*105 (7.2)*
Patients not up to date – n (%)	823 (47.6)	578 (40.1)
- *Contraindications to screening, CRC symptoms or risk factors in patients not up to date – n (%)*	*131 (7.6)*	*120 (8.3)*
Missing information on CRC screening status – n (%)	76 (4.4)	80 (5.5)
**Discussion on screening among patients without previous screening and no contraindications to screening – N (%)**	**768 (100)**	**538 (100)**
CRC screening discussed – n (%)	348 (46.8)	224 (43.9)
CRC not discussed – n (%)	420 (54.7)	314 (58.4)
- *Situation not suited – n (%)*	*248 (33.4)*	*188 (36.9)*
- *CRC previously discussed – n (%)*	*66 (8.9)*	*45 (8.8)*
- *Data already collected on this patient – n (%)*	*17 (2.3)*	*14 (2.8)*
- *Other/missing – n (%)*	*89 (11.6)*	*67 (12.5)*
**Patient’s decision after discussion – N (%)**	**348 (100)**	**224 (100)**
Test planned – n (%)	187 (53.7)	123 (54.9)
Refused testing – n (%)	125 (35.9)	73 (32.6)
Missing/no decision – n (%)	36 (10.3)	28 (12.5)
**Test planned (colonoscopy, FOBT or other) – N (%)**	**187 (100)**	**123 (100)**
Colonoscopy – n (%)	124 (66.3)	48 (39.0)
FOBT – n (%)	60 (32.1)	75 (61.0)
Other – n (%)	3 (1.6)	0 (0)
**Refused testing – N (%)**	**125 (100)**	**73 (100)**
Did not feel at risk of CRC – n (%)	53 (42.4)	50 (68.5)
Fear of adverse effects of test – n (%)	21 (16.8)	10 (13.7)
Financial barrier – n (%)	3 (2.4)	0 (0)
Other – n (%)	29 (23.2)	5 (6.9)
No reason given – n (%)	19 (15.2)	8 (11.0)

N indicates the total number of patients per randomized group. n indicates the number of patients within the specified subgroup.

Up-to-date CRC screening rate in 2018 (FOBT< 2 years or colonoscopy <10 years) at PCP visit was 50.9% (1,614/3171 patients); screening rates were higher in the intervention than in the control group before the discussion (54.4% vs. 48.0%; RR:1.13; 95% CI: 1.06 to 1.21, see Table 2). In 2017 collected data, this difference was already apparent when restricting the sample to PCP who collected data in 2017 and 2018 (see S5 Table).

## Discussion

In a context where colonoscopy is more used than FOBT for CRC screening such as in Swiss primary care, a mailed training intervention for PCP apparently increased both the number of PCP whose patients were prescribed FOBT for CRC screening and the number of PCP who intended to prescribe FOBT in primary analyses. However, when restricting analyses to PCP who participated in data collection in 2017, before the intervention took place, we found an apparent imbalance in distribution of PCP who already met the primary outcome and selective attrition of PCP in the intervention group, which suggest selection bias of PCP who participated in the outcome data collection in 2018. PCP who were already prescribing FOBT and had high CRC screening rates overall in 2017 tended to participate in the outcome data collection in 2018 more than those with no FOBT screening or low CRC screening rate in 2017. Since we found no change in prescription for FOBT within participating PCP over time, our results suggest no effect of the intervention on the number of PCP whose patients were prescribed FOBT for CRC screening. The intervention may still have increased PCP’s intentions to prescribe FOBT, a secondary outcome. We found that a higher number of patients had a FOBT planned after discussion in the intervention group compared to the control group at an aggregate, patient-level (Table 3). The analyses restricted to patient included by PCP having participated in both data collections confirmed this finding (S5 Table). Since we didn’t find in increase in the main outcome, but a slight increase in the proportion of PCP whose proportion of patients tested before the clinical visit or planning to be tested during the visit with FOBT vs colonoscopy was at least 40% (S4 Table), this increase in patients who had a FOBT planned after discussion is possibly due to PCP already prescribing FOBT prescribing it to a higher proportion of their patients after discussion in the intervention group in 2018 compared to 2017. The limited sample size and the chosen main outcome might also have led us to fail to detect clinically significant positive effects of the intervention on PCP prescription behavior.

While our main analysis suggests improved FOBT screening rates, our PCP-level mailed intervention probably did not improve the proportion of PCP prescribing FOBT. An earlier study conducted in the US found that offering a choice of FOBT and colonoscopy, compared to colonoscopy alone or FOBT alone, increased FOBT rates and overall screening rates compared to colonoscopy alone [11]. In contrast to our intervention, based on simply mailing the intervention to PCP and aiming at having PCP implement the proposed changes, that prior study enrolled PCP willing to consequently change their prescription practice according to the group they were randomized in. While PCP consented to data collection and participation in our trial, we did not only select PCP willing to change their prescription practices, but PCP interested in improving CRC screening rates overall. They also belonged to a nationwide Surveillance Network and were well trained in data collection. We believe researchers interested in improving CRC screening rates should be mindful of the target population they want to intervene on. Also, outcome measure should not rely on PCP willingness to collect data, but should instead rely on passively collected outcomes, such as administrative claims data. In an earlier analysis of data collected among the same PCP in 2017, CRC screening rates were 69% in patients whose PCP offered a choice of FOBT and colonoscopy but dropped to 38% for PCP who offered only colonoscopy; patients offered only colonoscopy had higher refusal rates. Our intention was to encourage prescription of FOBT in addition to colonoscopy, which could have increased FOBT prescription rates and thus overall screening rates through lower refusal rates. While our main analyses suggest we were able to increase the proportion of PCP who prescribed FOBT, selection bias of PCP participating in 2018 data collection is a more likely explanation. We stratified randomization based on PCP participation in 2017 data collection but not based on their prescription behaviour in 2017. While we did not find significant differences in proportion of PCP who already prescribed FOBT in 2017 between groups, a trend in this direction was already apparent. With low number of randomized groups, such imbalances due to chance are unavoidable. This imbalance might have been exacerbated by selective attrition in the intervention group. Fewer PCP participated in 2018 data collection in the intervention group compared to the control group (7 less; 38 vs 45). Some PCP in the intervention group who disagree with prescribing FOBT for CRC screening might have decided not to collect data when receiving the intervention package. In our case, even if number of PCP not participating was small, selective attrition of PCP not prescribing FOBT in the intervention group might have further driven the results towards an apparent significant effect. We believe the null findings, i.e., the absence of detectable effects of the intervention the proportion of PCP prescribing at least one FOBT is confirmed by the analyses on change in prescription behaviour within PCP and between the two data collections in the restricted sample (S3 Table). However, we still found an increase in intention of PCP to prescribe FOBT. Further, more patients of PCP in the intervention group chose FOBT after discussion, than patients in the control group. These findings align with results from a pilot study where an in-person educational training program for PCP was observed to lead to an increase in PCP’ intentions to prescribe FOBT [17]. The few months between the time the intervention package was sent and the data collection is another explanation of the null finding. Had we allowed more time for PCP to implement the intended changes, maybe we could have detected a significant effect of the intervention on the primary outcome.

Even though patient-centered interventions conducted in other countries were successful, we targeted PCP and did not survey patients directly [11,20–22]. We took this approach because we were interested in reducing PCP barriers to implementing shared decision-making about CRC screening, as described in recent systematic reviews [23,24]. We intended to lower these barriers through a multi-component intervention that targeted PCP, by ensuring our consultation material was effective, and by using a data collection form that had been created by PCP, who had ensured the form was clear and easy to integrate into PCP’ often busy daily routine. This increased applicability and possible sustainability of our training material. We drew on multiyear development and adaptation of our tested training material, including patient decision aids, where we applied principles of community-engaged and participatory research, involving an interprofessional group of physicians, pharmacists, public health experts, stakeholders and citizens [16–18,25]. Physicians who previously participated in our 2017 data collection were given personalized performance feedback, which can alert PCP to the dissonance of their perceived performance and their actual performance concerning CRC screening. Despite this extensive upfront work, our intervention most probably failed to change prescription behavior of PCP. In parallel to this rather passive intervention, our team conducted a RCT testing the effect of discussing the developed material within quality circles of physicians, a much more intensive intervention to change screening behavior of PCP. This trial included further PCP in Switzerland not participating in Sentinella. Analyses of the results of this trial are ongoing.

Our study has limitations. PCP may have studied and applied the material we provided to different degrees. Since we did not validate the data by checking patient charts, we could not determine the accuracy of PCP self-reports. Since we could not verify consecutive inclusion of patients, PCP could have introduced selection bias. But such bias is unlikely because our CRC screening rates align with our findings from a 2017 nationwide health survey and analyses of claims data from a large Swiss health insurance [10,26]. Further self-selection bias could have been introduced by PCP who participated in 2017 and did not prescribe FOBT before choosing to opt out of the study in 2018.- As written above, we believe self-selection bias at the time of randomization and selective attrition of PCP in the intervention group compared to the control group are the most likely explanations for the apparent effectiveness of the intervention. In addition, further bias might explain the apparent positive effects: Because PCP were aware of their assignment, PCP in the intervention group could have overreported true FOBT rates to please the research team, possibly leading us to overestimate the effect of the intervention. Finally, because we could not ask patients about their preferences or whether they felt their PCP used a SDM approach during the consultation, we cannot be sure patients chose the CRC test that best matched their preferences and values. While simply offering more than one screening option may influence uptake, SDM additionally involves an explicit discussion of benefits, harms, and patient values, which we could not measure directly. We decided not to add verification mechanisms to assess the use of the intervention material or SDM implementation for three reasons. First, this was a pragmatic trial that included busy PCP and adding controlling measures would likely have reduced the number of PCP who were interested in participating. Second, our study design prevented us from identifying enrolled patients so we could not assess SDM at the patient level. Third, the tools for assessing SDM implementation are not reliable, since they mostly rely on physician- or patient-reported data. Instead, we used a proxy for SDM, positing that if none of a PCP’s 40 consecutive patients was prescribed FOBT, it was highly unlikely that patients and doctors had shared the decision about which test to choose, and likely that the PCP offered or promoted only colonoscopy.

## Conclusion

In a country where colonoscopy is the most prescribed method for CRC screening, a mailed intervention that promoted a choice of screening tests in primary care appeared to increase the number of PCP who prescribed FOBT. However, selective attrition in the intervention group likely drove this apparent effect and our sample sizes were rather low. The intervention mainly increased the intention of PCP already prescribing FOBT to prescribe FOBT to even more patients, a secondary outcome. These findings should encourage researchers to test the intervention material in different contexts of care, with a larger sample of PCP randomized, stratified for their prescription patterns and to repeat data collection over a larger time-span to capture sustainability and effect on actual prescription patterns. Future studies should also enrich the data collection with patient-reported outcomes on their level of understanding of the two screening options and attitudes towards these.

## Supporting information

S1 Table PCP characteristics (PCP age, sex, language, area of practice and patient age and sex) 2017 at randomization for trial in 2018. (DOCX)

S2 Table Characteristics of PCP (age, sex, language, area of practice and patient age and sex) having initially agreed to participate in the RCT and randomized into control and intervention group – intention to treat analysis. (DOCX)

S3 Table Sensitivity analysis for primary outcome of proportion of PCP who had at least one patient previously tested with FOBT, or who prescribed at least one FOBT to eligible patients during the data collection period. (DOCX)

S4 Table Changes in primary outcome and one secondary outcome between PCP participating both in 2017 and 2018 during the data collection period. (DOCX)

S5 Table Details on discussion about CRC screening by trial arm, stratified by data collected in 2017 and in 2018 and restricted to PCP having participated in both data collections. (DOCX)

S1 Fig Data collection form: used to collect all outcomes. (PDF)

S2 Fig Flow Chart Data collecting: a flow chart describing how the data collection form is supposed to be filled out. (PDF)

S3 Fig Decision Box: Two pages filled with current guidelines and evidence on colorectal cancer incidence and prevalence in Switzerland, Flowcharts explaining with total numbers the sensitivity and specificity of FIT and colonoscopy and further screening plan after results are obtained. Also, risks and benefits of each test are listed with sources on which evidence these assessments are based on.
(PDF)

S4 Fig Decision Board: Two laminated sheets of paper, printed with well-visualized information for PCP to inform patients about the prevalence and mortality rate of colorectal cancer, where and how it is formed in the body, the two screening types available and recommended by the guidelines (FOBT and colonoscopy) and how the screening with them is performed, and lastly a table listing both screening methods and comparing them in terms of costs, risks and benefits and accuracy of the test results. (PDF)

S5 Fig Example of personal feedback from 2017: Two sheets of paper printed with well visualized information for PCP to inform them about how the screening rates of their patients and the chosen methods for this screening compared to the rest of the physicians in the cohort of 91 PCP in our data collection in 2017. (PDF)

S6 Fig Modified primary outcome: Proportion of PCP with at least one patient tested with FOBT before discussion (without considering FOBT prescribed during the data collection period). (PDF)

S1 File CONSORT checklist: Filled out CONSORT checklist based on our study specifics. (PDF)

## Abbreviations

FOBT: fecal occult blood test
CRC: colorectal cancer
PCP: primary care physicians
SDM: shared decision-making
FOPH: Federal Office of Public Health
