# Supplementary material for: Promoting shared decision-making in colorectal cancer screening in primary care: A cluster randomized controlled trial
Source: PLoS One. 2026 Jun 9;21(6):e0351069. doi: 10.1371/journal.pone.0351069 (PMC13249137; doi:10.1371/journal.pone.0351069)
Supplement: S1 Table — (DOCX) [file pone.0351069.s001.docx]

**S1 Table. PCP characteristics 2017 at randomization for trial in 2018**

| **PCP characteristics** | **Control (N = 37)** | **Intervention (N = 37)** |
| --- | --- | --- |
| Age, mean* | 56.4 | 55.2 |
| Women - n (%) | 5 (13.5) | 11 (29.7) |
| Language region |  |  |
| German - n (%) | 27 (73.0) | 24 (64.9) |
| French - n (%) | 7 (18.9) | 11 (29.7) |
| Italian - n (%) | 3 (8.1) | 2 (5.4) |
| Area of practice |  |  |
| Urban - n (%) | 24 (64.9) | 29 (78.4) |
| Intermediate - n (%) | 6 (16.2) | 7 (18.9) |
| Rural - n (%) | 7 (18.9) | 1 (2.7) |
| **Patient characteristics** | **Control (N = 1,402)** | **Intervention (N = 1,399)** |
| Age – mean | 62.6 | 62.7 |
| Women - n (%) | 679 (48.4) | 740 (52.9) |

N indicates the total number of physicians/patients per randomized group. n indicates the number of physicians/patients within the specified subgroup.

*Mean age of PCPs was estimated from grouped age ranges using midpoints of each age group; results should be interpreted as approximate.
