# Supplementary material for: Promoting shared decision-making in colorectal cancer screening in primary care: A cluster randomized controlled trial
Source: PLoS One. 2026 Jun 9;21(6):e0351069. doi: 10.1371/journal.pone.0351069 (PMC13249137; doi:10.1371/journal.pone.0351069)
Supplement: S2 Table — (DOCX) [file pone.0351069.s002.docx]

**S2 Table. Characteristics of PCP having initially agreed to participate in the RCT and randomized into control and intervention group – intention to treat analysis**

| **PCP characteristics** | **Control (N = 54)** | **Intervention (N = 56)** |
| --- | --- | --- |
| Age groups |  |  |
| 30-39 - n (%) | 6 (11.1) | 5 (8.9) |
| 40-49 - n (%) | 9 (16.7) | 15 (26.8) |
| 50-59 - n (%) | 16 (29.6) | 17 (30.4) |
| >60 - n (%) | 23 (42.6) | 19 (33.9) |
| Women - n (%) | 11 (20.4) | 15 (26.8) |
| Language region |  |  |
| German - n (%) | 37 (68.5) | 32 (57.1) |
| French - n (%) | 11 (20.4) | 19 (33.9) |
| Italian - n (%) | 6 (11.1) | 5 (8.9) |
| Area of practice |  |  |
| Urban - n (%) | 36 (66.7) | 40 (71.4) |
| Intermediate - n (%) | 8 (14.8) | 12 (21.4) |
| Rural - n (%) | 10 (18.5) | 4 (7.1) |

N indicates the total number of physicians per randomized group. n indicates the number of physicians within the specified subgroup.
