# Supplementary material for: Promoting shared decision-making in colorectal cancer screening in primary care: A cluster randomized controlled trial
Source: PLoS One. 2026 Jun 9;21(6):e0351069. doi: 10.1371/journal.pone.0351069 (PMC13249137; doi:10.1371/journal.pone.0351069)
Supplement: S3 Table — (DOCX) [file pone.0351069.s003.docx]

**S3 Table. Sensitivity analysis for primary outcome of proportion of PCP who had at least one patient previously tested with FOBT, or who prescribed at least one FOBT to eligible patients during the data collection period**

| Analyses by randomized groups (intervention vs control) | Control | Intervention | Relative Risk (95% CI),  p-value |
| --- | --- | --- | --- |
| Main outcome in 2017, analyses restricted to PCP who participated in 2017 and were randomized in 2018 - % (n/N) | 51.4 (19/ 37) | 64.9 (24/37) | 1.26 (95% CI: 0.85-1.88), p=0.25 |
| Main outcome in 2017, analyses restricted to PCP who participated in 2017 and 2018 - % (n/N) | 48.5 (16/33) | 72.4 (21/29) | 1.49 (95% CI: 0.98-2.28), p=0.06 |
| Main outcome in 2018, analyses restricted to PCP who participated in 2017 and 2018 - % (n/N) | 54.5 (18/33) | 82.8 (24/29) | 1.52 (95% CI: 1.06-2.17), p=0.02 |
| Main outcome in 2018 with included PCP who also participated in 2017 and were randomized but did not participate, assuming no change in FOBT screening rates - % (n/N) | 56.3 (27/48) | 75.6 (34/45) | 1.34 (95% CI: 0.99-1.82), p=0.06 |
|  |  |  |  |
| Analyses by year of outcome assessment (2017 vs 2018, data from PCP intervention and control group merged) | 2017 | 2018 | Relative Risk (95% CI), p-value |
| Main outcome compared between PCP data reporting in 2017 and PCP data reporting in 2018 - % (n/N) | 58.2 (53/91) | 68.7 (57/83) | 1.18 (95% CI: 0.94-1.48), p=0.16 |
| Main outcome compared between 2017 and 2018 for PCP participating in both years - % (n/N) | 59.7 (37/62) | 67.7 (42/62) | 1.14 (95% CI: 0.87-1.48), p=0.35 |

N indicates the total number of physicians per randomized group. n indicates the number of physicians within the specified subgroup.
