# Supplementary material for: Promoting shared decision-making in colorectal cancer screening in primary care: A cluster randomized controlled trial
Source: PLoS One. 2026 Jun 9;21(6):e0351069. doi: 10.1371/journal.pone.0351069 (PMC13249137; doi:10.1371/journal.pone.0351069)
Supplement: S4 Table — (DOCX) [file pone.0351069.s004.docx]

**S4 Table. Changes in primary outcome between PCP participating both in 2017 and 2018 during the data collection period**

|  | Data | 2017 | Data | 2018 |
| --- | --- | --- | --- | --- |
|  | Control | Intervention | Control | Intervention |
| PCP who had at least one patient previously tested with FOBT, or who prescribed at least one FOBT to eligible patients - % (n/N) | 57.6 (19/33) | 72.4 (21/29) | 63.6 (21/33) | 82.8 (24/29) |
| Started prescribing at least one FOBT during the data collection period in 2018 -  % (n/N) | N/A | N/A | 21.2 (7/33) | 13.8 (4/29) |
| Stopped prescribing at least one FOBT during the data collection period in 2018 -  % (n/N) | N/A | N/A | 15.2 (5/33) | 3.5 (1/29) |
| PCP whose proportion of patients tested before the clinical visit or planning to be tested during the visit with FOBT vs colonoscopy was at least 40% - % (n/N) | 39.4 (13/33) | 55.2 (16/29) | 39.4 (13/33) | 62.1 (18/29) |

N indicates the total number of physicians per randomized group. n indicates the number of physicians within the specified subgroup.
