# Supplementary material for: Promoting shared decision-making in colorectal cancer screening in primary care: A cluster randomized controlled trial
Source: PLoS One. 2026 Jun 9;21(6):e0351069. doi: 10.1371/journal.pone.0351069 (PMC13249137; doi:10.1371/journal.pone.0351069)
Supplement: S5 Table — (DOCX) [file pone.0351069.s005.docx]

**S5 Table. Details on discussion about CRC screening by trial arm, stratified by data collected in 2017 and in 2018 and restricted to PCP having participated in both data collections.**

|  | **Data** | **2017** | **Data** | **2018** |
| --- | --- | --- | --- | --- |
|  | **Control** | **Intervention** | **Control** | **Intervention** |
| **Total patients included – N (%)** | **1251 (100)** | **1089 (100)** | **1267 (100)** | **1107 (100)** |
| Patients up to date with CRC testing before discussion - n (%) | 562 (44.9) | 524 (48.1) | 632 (49.9) | 602 (54.4) |
| - *Colonoscopy - n (%)* | *501 (40.0)* | *482 (44.3)* | *559 (44.1)* | *518 (46.8)* |
| - *FOBT/FIT - n (%)* | *61 (4.9)* | *42 (3.9)* | *73 (5.8)* | *84 (7.6)* |
| Patients not up to date - n (%) | 627 (50.1) | 525 (48.2) | 582 (45.9) | 446 (40.3) |
| - *Contraindications to screening in patients not up to date - n (%)* | *100 (8.0)* | *80 (7.3)* | *85 (6.7)* | *96 (8.7)* |
| Missing information on CRC screening status - n (%) | 62 (5.0) | 40 (3.7) | 53 (4.2) | 59 (5.3) |
| **Discussion on screening among patients without previous screening and no contraindications to screening - N (%)** | **589 (100)** | **485 (100)** | **550 (100)** | **409 (100)** |
| CRC screening discussed - n (%) | 297 (50.4) | 224 (46.2) | 259 (47.1) | 170 (41.6) |
| CRC not discussed - n (%) | 292 (49.6) | 261 (53.8) | 291 (52.9) | 239 (58.4) |
| - *Situation not suited - n (%)* | *203 (34.5)* | *159 (32.8)* | *163 (29.6)* | *142 (34.7)* |
| - *CRC previously discussed - n (%)* | *31 (5.3)* | *38 (7.8)* | *56 (10.2)* | *32 (7.8)* |
| - *Data already collected on this patient - n (%)* | *0 (0.0)* | *0 (0.0)* | *9 (1.6)* | *6 (1.5)* |
| - *Other/missing - n (%)* | *58 (9.8)* | *64 (13.2)* | *63 (11.5)* | *59 (14.4)* |
| **Patient’s decision after discussion - N (%)** | **297 (100)** | **224 (100)** | **259 (100)** | **170 (100)** |
| Test planned - n (%) | 163 (54.9) | 146 (65.2) | 139 (53.7) | 96 (56.5) |
| Refused testing - n (%) | 101 (34.0) | 66 (29.5) | 90 (34.7) | 50 (29.4) |
| Missing/no decision - n (%) | 33 (11.1) | 12 (5.4) | 30 (11.6) | 24 (14.1) |
| **Test planned (colonoscopy, FOBT or other) - N (%)** | **163 (100)** | **146 (100)** | **139 (100)** | **96 (100)** |
| Colonoscopy - n (%) | 98 (60.1) | 72 (49.3) | 87 (62.6) | 33 (34.4) |
| FOBT - n (%) | 65 (39.9) | 73 (50.0) | 50 (36.0) | 63 (65.6) |
| Other - n (%) | 0 (0.0) | 1 (0.7) | 2 (1.4) | 0 (0.0) |
| **Refused testing - N (%)** | **101 (100)** | **66 (100)** | **90 (100)** | **50 (100)** |
| Did not feel at risk of CRC - n (%) | 21 (20.8) | 1 (1.5) | 11 (12.2) | 6 (12.0) |
| Fear of adverse effects of test - n (%) | 44 (43.6) | 41 (62.1) | 38 (42.2) | 35 (70.0) |
| Financial barrier - n (%) | 11 (10.9) | 10 (15.2) | 16 (17.8) | 4 (8.0) |
| Other - n (%) | 2 (2.0) | 3 (4.5) | 1 (1.1) | 0 (0.0) |
| No reason given - n (%) | 23 (22.8) | 11 (16.7) | 24 (26.7) | 5 (10.0) |

N indicates the total number of patients per randomized group. n indicates the number of patients within the specified subgroup.
