# Supplementary material for: Promoting shared decision-making in colorectal cancer screening in primary care: A cluster randomized controlled trial
Source: PLoS One. 2026 Jun 9;21(6):e0351069. doi: 10.1371/journal.pone.0351069 (PMC13249137; doi:10.1371/journal.pone.0351069)
Supplement: S1 Fig — (PDF) [file pone.0351069.s006.pdf]

S1 Fig. Data collection form

SENTINELLA

Data collection form Colorectal cancer screening 2017

Physician code ☐☐☐☐ **Sheet N°** ☐

Method of **consecutively** including Patients ☐
1 = Strict consecutively
2 = First two Paients per half-day

Planned colorectal cancer screening	**1** = No screening planned- **2** = Colonoscopy - **3** = FOBT* - **4** = Other tests- **9** = Missing data
Reason of refusal of patient	**1** = No refusal- **2** = No reason- **3** = Patient doesn't feel concerned - **4** = Fear of sideeffects or complications - **5** = Financial barriers- **6** = Other reason- **9** = Missing data
Riskfactors for colorectal cancer	**1** = No riskfactors for colorectal cancer- **2** = Personal history of colorectal cancer or polyps- **3** = Family history of colorectal cancer or polyps - **4** = History of M. Chron or colitis ulcerosa- **5** = Other riskfactors - **9** = Missing data
Symptoms of colorectal cancer	**1** = No symptoms of colorectal cancer- **2** = Symptoms suggesting coorectal cancer (blood in stool, stomach pain, weightloss, changes in digestion, etc.) - **9** = Missing data
	STOP: if 2 , 3, 4 or 5
Talk about colorectal cancer screening during consultation	**1** = Yes - **2** = Consultation inappropriate for a talk- **3** = No, a talk has already taken place - **4** = Patient already seen during this Data collection - **5** = Other medical reasons not to have a talk- **9** = Missing data
	STOP: if 2 , 3 or 4
Medical contra-indications for colorectal cancer screening	**1** = No medical contraindications - **2** = Life expectancy < 5 years - **3** = Severe acute Illness - **4** = Other contraindication - **9** = Missing data
	STOP: if 2 , 4 or 6
History of colorectal cancer screening	**1** = Never screened- **2** = Colondoscopy < 10 years- **3** = Colondoscopy > 10 years - **4** = FOBT* < 2 years - **5** = FOBT * > 2 years - **6** = Other test - **9** = Screening history unknown
Birthyear (between 1941 and 1967)	
Sex 1=male 2=female	
Week	
N° pat.	01 02 03 04 05 06 07 08 09 10 11 12 13 14 15 16 17 18 19 20

40 consecutive Patients 50-75 years old (born 1941 to 1967)

* To facilitate the data collection we will not distinguish between guaiac based or immunochemical tests (FIT)
