# Supplementary material for: Promoting shared decision-making in colorectal cancer screening in primary care: A cluster randomized controlled trial
Source: PLoS One. 2026 Jun 9;21(6):e0351069. doi: 10.1371/journal.pone.0351069 (PMC13249137; doi:10.1371/journal.pone.0351069)

S2 Fig. Flow Chart Data collecting

A flow chart describing how the data collection form is supposed to be filled out.

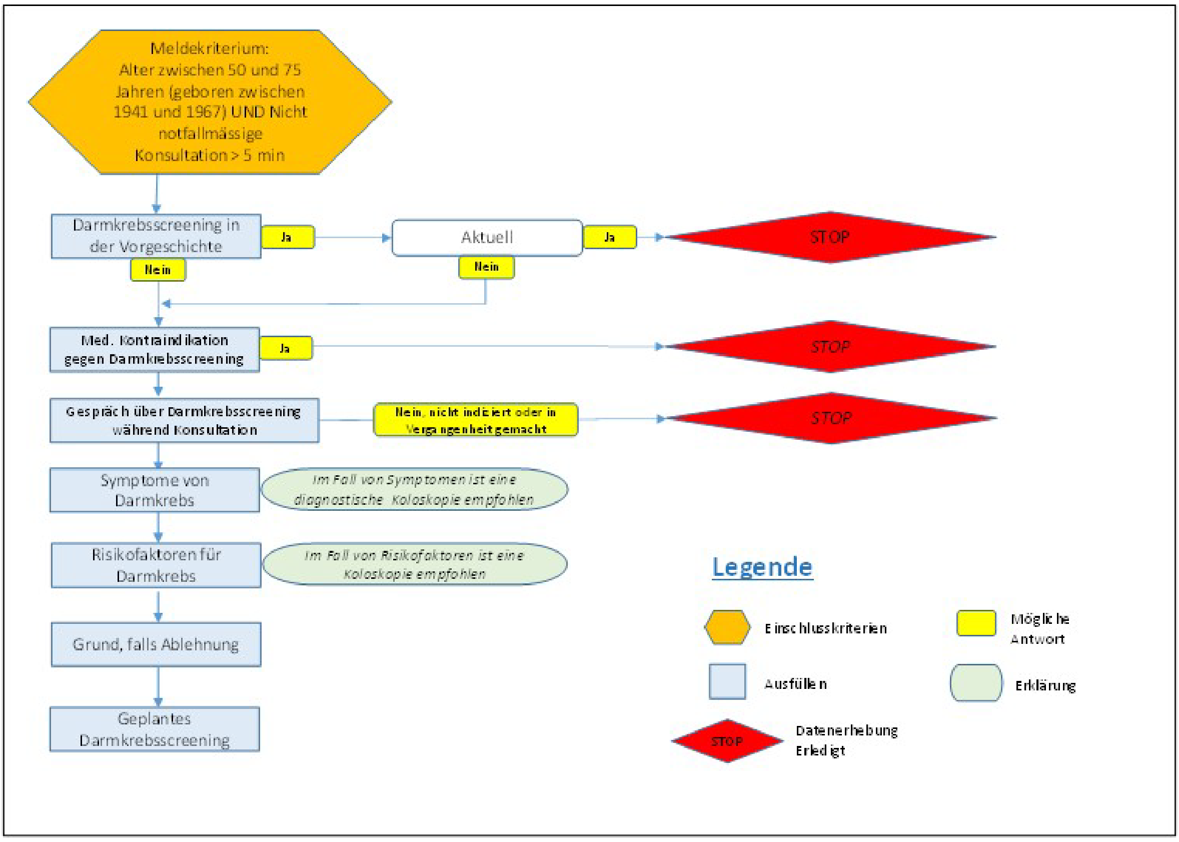

Supplement: S2 Fig — (PDF) [file pone.0351069.s007.pdf]
