# Supplementary material for: Promoting shared decision-making in colorectal cancer screening in primary care: A cluster randomized controlled trial
Source: PLoS One. 2026 Jun 9;21(6):e0351069. doi: 10.1371/journal.pone.0351069 (PMC13249137; doi:10.1371/journal.pone.0351069)
Supplement: S3 Fig — Also, risks and benefits of each test are listed with sources on which evidence these assessments are based on. (PDF) [file pone.0351069.s008.pdf]

S3 Fig. Decision Box

Two pages filled with current guidelines and evidence on colorectal cancer incidence and prevalence in Switzerland, Flowcharts explaining with total numbers the sensitivity and specificity of FIT and colonoscopy and further screening plan after results are obtained. Also, risks and benefits of each test are listed with sources on which evidence these assessments are based on.

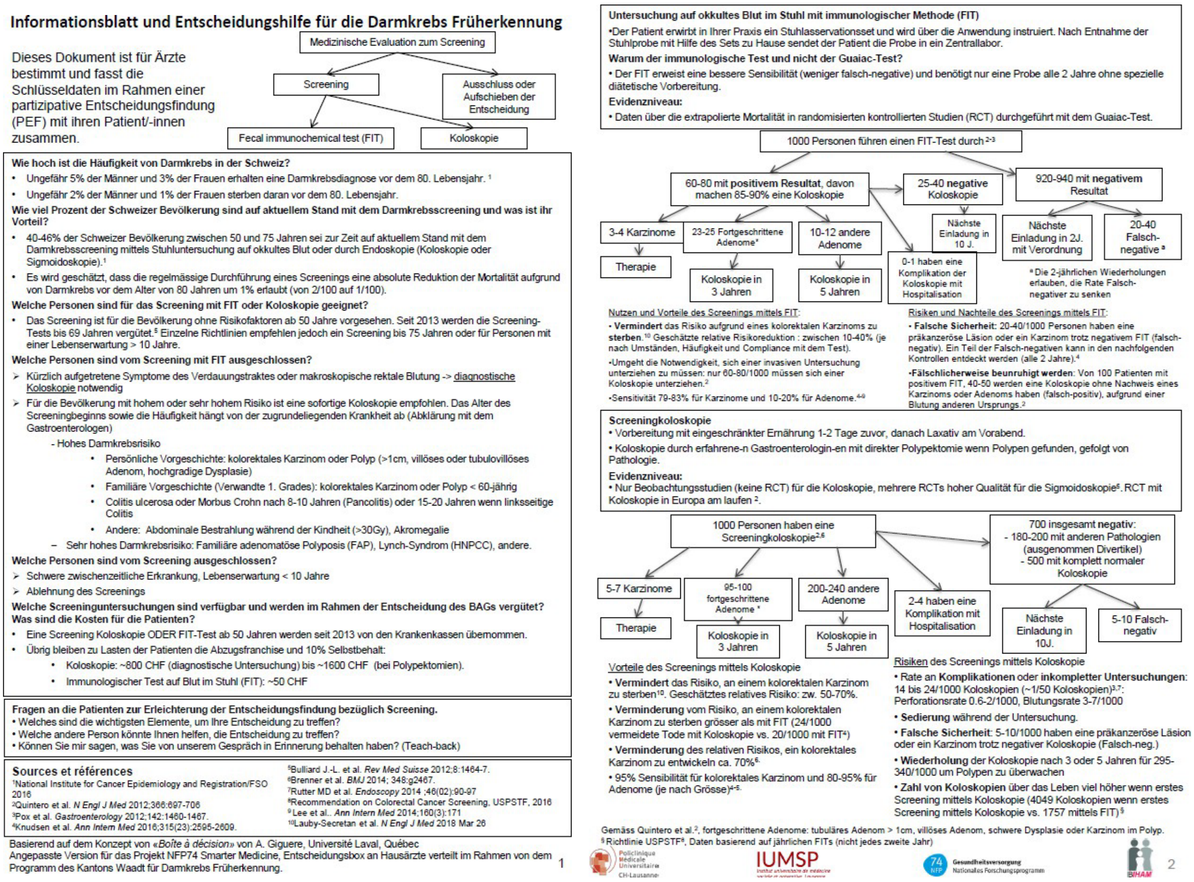

Informationsblatt und Entscheidungshilfe für die Darmkrebs Früherkennung

Dieses Dokument ist für Ärzte bestimmt und fasst die Schlüsseldaten im Rahmen einer partizipative Entscheidungsfindung (PEF) mit ihren Patient/-innen zusammen.

Wie hoch ist die Häufigkeit von Darmkrebs in der Schweiz?

- Ungefähr 5% der Männer und 3% der Frauen erhalten eine Darmkrebsdiagnose vor dem 80. Lebensjahr. [1]
- Ungefähr 2% der Männer und 1% der Frauen sterben daran vor dem 80. Lebensjahr.

Wie viel Prozent der Schweizer Bevölkerung sind auf aktuellem Stand mit dem Darmkrebsscreening und was ist ihr Vorteil?

- 40-46% der Schweizer Bevölkerung zwischen 50 und 75 Jahren sei zur Zeit auf aktuellem Stand mit dem Darmkrebsscreening mittels Stuhluntersuchung auf okkultes Blut oder durch Endoskopie (Koloskopie oder Sigmoidoskopie).[1]
- Es wird geschätzt, dass die regelmässige Durchführung eines Screenings eine absolute Reduktion der Mortalität aufgrund von Darmkrebs vor dem Alter von 80 Jahren um 1% erlaubt (von 2/100 auf 1/100).

Welche Personen sind für das Screening mit FIT oder Koloskopie geeignet?

- Das Screening ist für die Bevölkerung ohne Risikofaktoren ab 50 Jahre vorgesehen. Seit 2013 werden die Screening-Tests bis 69 Jahren vergütet.[5] Einzelne Richtlinien empfehlen jedoch ein Screening bis 75 Jahren oder für Personen mit einer Lebenserwartung > 10 Jahre.

Welche Personen sind vom Screening mit FIT ausgeschlossen?

- Kürzlich aufgetretene Symptome des Verdauungstraktes oder makroskopische rektale Blutung -> diagnostische Koloskopie notwendig
- Für die Bevölkerung mit hohem oder sehr hohem Risiko ist eine sofortige Koloskopie empfohlen. Das Alter des Screeningbeginns sowie die Häufigkeit hängt von der zugrundeliegenden Krankheit ab (Abklärung mit dem Gastroenterologen)
  - Hohes Darmkrebsrisiko
    - Persönliche Vorgeschichte: kolorektales Karzinom oder Polyp (>1cm, villöses oder tubulovillöses Adenom, hochgradige Dysplasie)
    - Familiäre Vorgeschichte (Verwandte 1. Grades): kolorektales Karzinom oder Polyp < 60-jährig
    - Colitis ulcerosa oder Morbus Crohn nach 8-10 Jahren (Pancolitis) oder 15-20 Jahren wenn linksseitige Colitis
    - Andere: Abdominale Bestrahlung während der Kindheit (>30Gy), Akromegalie
  - Sehr hohes Darmkrebsrisiko: Familiäre adenomatöse Polyposis (FAP), Lynch-Syndrom (HNPCC), andere.

Welche Personen sind vom Screening ausgeschlossen?

- Schwere zwischenzeitliche Erkrankung, Lebenserwartung < 10 Jahre
- Ablehnung des Screenings

Welche Screeninguntersuchungen sind verfügbar und werden im Rahmen der Entscheidung des BAGs vergütet? Was sind die Kosten für die Patienten?

- Eine Screening Koloskopie ODER FIT-Test ab 50 Jahren werden seit 2013 von den Krankenkassen übernommen.
- Übrig bleiben zu Lasten der Patienten die Abzugsfranchise und 10% Selbstbehalt:
  - Koloskopie: ~800 CHF (diagnostische Untersuchung) bis ~1600 CHF (bei Polypektomien).
  - Immunologischer Test auf Blut im Stuhl (FIT): ~50 CHF

Fragen an die Patienten zur Erleichterung der Entscheidungsfindung bezüglich Screening.

- Welches sind die wichtigsten Elemente, um Ihre Entscheidung zu treffen?
- Welche andere Person könnte Ihnen helfen, die Entscheidung zu treffen?
- Können Sie mir sagen, was Sie von unserem Gespräch in Erinnerung behalten haben? (Teach-back)

Sources et références

[1]National Institute for Cancer Epidemiology and Registration/FSO 2016
[2]Quintero et al. *N Engl J Med* 2012;366:697-706
[3]Pox et al. *Gastroenterology* 2012;142:1460-1467.
[4]Knudsen et al. *Ann Intern Med* 2016;315(23):2595-2609.
[5]Bulliard J.-L. et al. *Rev Med Suisse* 2012;8:1464-7.
[6]Brenner et al. *BMJ* 2014; 348:g2467.
[7]Rutter MD et al. *Endoscopy* 2014 ;46(02):90-97
[8]Recommendation on Colorectal Cancer Screening, USPSTF, 2016
[9]Lee et al.. *Ann Intern Med* 2014;160(3):171
[10]Lauby-Secretan et al. *N Engl J Med* 2018 Mar 26

Basierend auf dem Konzept von «*Boîte à décision*» von A. Giguere, Université Laval, Québec
Angepasste Version für das Projekt NFP74 Smarter Medicine, Entscheidungsbox an Hausärzte verteilt im Rahmen von dem Programm des Kantons Waadt für Darmkrebs Früherkennung. 1

Untersuchung auf okkultes Blut im Stuhl mit immunologischer Methode (FIT)

- Der Patient erwirbt in Ihrer Praxis ein Stuhlasservationsset und wird über die Anwendung instruiert. Nach Entnahme der Stuhlprobe mit Hilfe des Sets zu Hause sendet der Patient die Probe in ein Zentrallabor.

Warum der immunologische Test und nicht der Guaiac-Test?

- Der FIT erweist eine bessere Sensibilität (weniger falsch-negative) und benötigt nur eine Probe alle 2 Jahre ohne spezielle diätetische Vorbereitung.

Evidenzniveau:

- Daten über die extrapolierte Mortalität in randomisierten kontrollierten Studien (RCT) durchgeführt mit dem Guaiac-Test.

<u>Nutzen und Vorteile des Screenings mittels FIT</u>:

- **Vermindert** das Risiko aufgrund eines kolorektalen Karzinoms zu **sterben**.[10] Geschätzte relative Risikoreduktion : zwischen 10-40% (je nach Umständen, Häufigkeit und Compliance mit dem Test).
- Umgeht die Notwendigkeit, sich einer invasiven Untersuchung unterziehen zu müssen: nur 60-80/1000 müssen sich einer Koloskopie unterziehen.[2]
- Sensitivität 79-83% für Karzinome und 10-20% für Adenome.[4-9]

<u>Risiken und Nachteile des Screenings mittels FIT</u>:

- **Falsche Sicherheit:** 20-40/1000 Personen haben eine präkanzeröse Läsion oder ein Karzinom trotz negativem FIT (falsch-negativ). Ein Teil der Falsch-negativen kann in den nachfolgenden Kontrollen entdeckt werden (alle 2 Jahre).[4]
- **Fälschlicherweise beunruhigt werden**: Von 100 Patienten mit positivem FIT, 40-50 werden eine Koloskopie ohne Nachweis eines Karzinoms oder Adenoms haben (falsch-positiv), aufgrund einer Blutung anderen Ursprungs.[2]

Screeningkoloskopie

- Vorbereitung mit eingeschränkter Ernährung 1-2 Tage zuvor, danach Laxativ am Vorabend.
- Koloskopie durch erfahrene-n Gastroenterologin-en mit direkter Polypektomie wenn Polypen gefunden, gefolgt von Pathologie.

Evidenzniveau:

- Nur Beobachtungsstudien (keine RCT) für die Koloskopie, mehrere RCTs hoher Qualität für die Sigmoidoskopie[5]. RCT mit Koloskopie in Europa am laufen [2].

<u>Vorteile</u> des Screenings mittels Koloskopie

- **Vermindert** das Risiko, an einem kolorektalen Karzinom zu sterben[10]. Geschätztes relatives Risiko: zw. 50-70%.
- **Verminderung** vom Risiko, an einem kolorektalen Karzinom zu sterben grösser als mit FIT (24/1000 vermeidete Tode mit Koloskopie vs. 20/1000 mit FIT[4])
- **Verminderung** des relativen Risikos, ein kolorektales Karzinom zu entwickeln ca. 70%[6].
- 95% Sensibilität für kolorektales Karzinom und 80-95% für Adenome (je nach Grösse)[4-5].

<u>Risiken</u> des Screenings mittels Koloskopie

- Rate an **Komplikationen** oder **inkompletter Untersuchungen**: 14 bis 24/1000 Koloskopien (~1/50 Koloskopien)[3,7]: Perforationsrate 0.6-2/1000, Blutungsrate 3-7/1000
- **Sedierung** während der Untersuchung.
- **Falsche Sicherheit**: 5-10/1000 haben eine präkanzeröse Läsion oder ein Karzinom trotz negativer Koloskopie (Falsch-neg.)
- **Wiederholung** der Koloskopie nach 3 oder 5 Jahren für 295-340/1000 um Polypen zu überwachen
- **Zahl von Koloskopien** über das Leben viel höher wenn erstes Screening mittels Koloskopie (4049 Koloskopien wenn erstes Screening mittels Koloskopie vs. 1757 mittels FIT)[§]

Gemäss Quintero et al.[2], fortgeschrittene Adenome: tubuläres Adenom > 1cm, villöses Adenom, schwere Dysplasie oder Karzinom im Polyp.
[§]Richtlinie USPSTF[8], Daten basierend auf jährlichen FITs (nicht jedes zweite Jahr)

Policlinique Médicale Universitaire CH-Lausanne · IUMSP · 74 NFP Gesundheitsversorgung Nationales Forschungsprogramm · BIHAM 2
