# Supplementary material for: Promoting shared decision-making in colorectal cancer screening in primary care: A cluster randomized controlled trial
Source: PLoS One. 2026 Jun 9;21(6):e0351069. doi: 10.1371/journal.pone.0351069 (PMC13249137; doi:10.1371/journal.pone.0351069)
Supplement: S4 Fig — (PDF) [file pone.0351069.s009.pdf]

S4 Fig. Decision Board

Two laminated sheets of paper, printed with well visualized information for PCP to inform patients about the prevalence and mortality rate of colorectal cancer, where and how it is formed in the body, the two screening types available and recommended by the guidelines (FOBT and colonoscopy) and how the screening with them is performed, and lastly a table listing both screening methods and comparing them in terms of costs, risks and benefits, and accuracy of the test results.

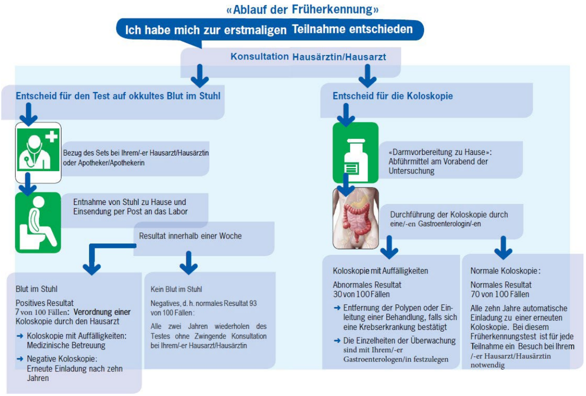

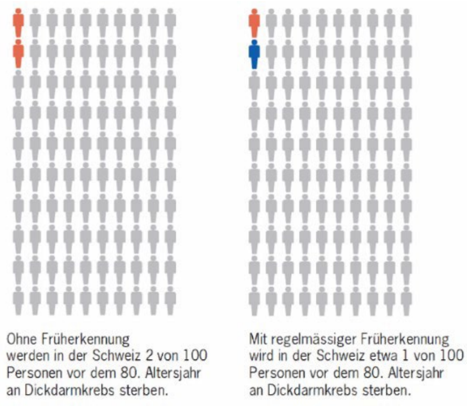

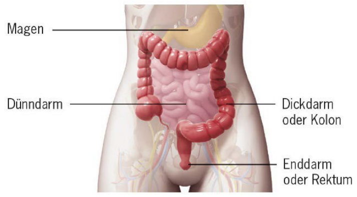

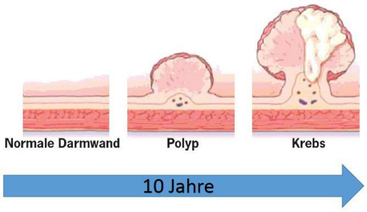

Durchführung – Vorteile, Risiken und Nachteile der Früherkennungstests

	Nachweis von okkultem Blut im Stuhl	Früherkennungskoloskopie
Wie wird der Test durchgeführt?	➜ Zu Hause Entnahme einer kleinen Menge Stuhl mit Hilfe einer speziell abgegebenen Vorrichtung<br>➜ Einsenden der Stuhlprobe per Post und Analyse in einem spezialisierten Labor	➜ «Vorbereitung zu Hause»: Diät während 48 Stunden und Einnahme einer speziellen Lösung zur «Reinigung» des Darms am Vorabend<br>➜ Durchführung in der Praxis eines Gastroenterologen, in einer Klinik oder einem Spital<br>➜ In der Regel erhalten Sie ein Beruhigungsmittel, das Sie in einen leichten Schlaf versetzt<br>➜ Die Untersuchung dauert gewöhnlich 30-45 Minuten oder, wenn Polypen entfernt werden müssen, manchmal auch länger
Erhalt der Resultate	➜ Information durch Ihren/e Hausarzt/Hausärztin	➜ Information durch Ihren/e Hausarzt/Hausärztin
Kostenübernahme	➜ Ab einem Alter von 50 Jahren wird die Stuhluntersuchung von den Krankenkassen übernommen. Übrig bleiben zu Lasten des Patienten die Abzugsfranchise und 10% Selbstbehalt ~ 46 CHF	➜ Ab einem Alter von 50 Jahren wird die Koloskopie von den Krankenkassen übernommen. Übrig bleiben zu Lasten des Patienten die Abzugsfranchise und 10% Selbstbehalt ~ 800-1600CHF
Häufigkeit der Durchführung	➜ **Alle zwei Jahre**	➜ **Alle zehn Jahre**
Welche Vorteile bestehen?	➜ Keine Vorbereitung notwendig<br>➜ Einfach durchzuführen	➜ Bester Test, um Polypen zu erkennen, bevor daraus Krebs entsteht<br>➜ Die Polypen werden während der Untersuchung direkt entfernt
Verlässlichkeit des Nachweises von Dickdarmkrebs	➜ Hoch, sofern der Test regelmässig alle zwei Jahre durchgeführt wird	➜ Sehr hoch, sofern die «Vorbereitung zu Hause» korrekt durchgeführt wird
Welche Nachteile bestehen?	➜ Risiko von «falsch negativen» Resultaten: Polypen und Tumoren bluten nicht immer. Der Test muss deshalb mindestens alle zwei Jahre wiederholt werden<br>➜ Risiko von «falsch positiven» Resultaten: Eine Blutung im Magen-Darm-Trakt kann auch auftreten, ohne dass Polypen oder Krebs vorliegen<br>➜ Bei einem positiven Test **muss systematisch eine Koloskopie durchgeführt werden**	➜ Sehr geringes Risiko einer schweren Komplikation (Darmperforation oder starke Blutung): etwa 2 Fälle auf 1`000 Koloskopien<br>➜ Falls Ihnen ein Beruhigungsmittel verabreicht wird, dürfen Sie während 12 bis 24 Stunden kein Fahrzeug lenken (Wirkung des Beruhigungsmittels)
