# Supplementary material for: Promoting shared decision-making in colorectal cancer screening in primary care: A cluster randomized controlled trial
Source: PLoS One. 2026 Jun 9;21(6):e0351069. doi: 10.1371/journal.pone.0351069 (PMC13249137; doi:10.1371/journal.pone.0351069)
Supplement: S5 Fig — (PDF) [file pone.0351069.s010.pdf]

S5 Fig. Example of personal feedback from 2017

Two sheets of paper, printed with well-visualized information for PCP to inform them about how the screening rates of their patients and the chosen methods for this screening compared to the rest of the physicians in the cohort of 91 PCP in our data collection in 2017.

Persönliches Feedback zu Ihren Darmkrebs-Screening-Praktiken in 2017

Um Sie einen Überblick von Ihrem Vorgehen bezüglich Darmkrebs-Screening zu zeigen, finden Sie auf dieser Seite verschiedene Ergebnisse, die Ihre Daten vom Ablauf zum Darmkrebs-Screening im Jahr 2017 mit den anderen Sentinella-Ärzten vergleichen. Diese Analysen basieren auf den 40 Patienten die Sie in 2017 in der Datenerhebung eingeschlossen haben. Angesichts einer kleinen Stichprobengrösse in jeder Praxis sind diese Anteile indikativ. (Gesamtzahl der Ärzte mit analysierten Daten = 91)

Abbildung 1 – Prozentsatz der Patienten die ein Darmkrebscreening hatten gemäss Empfehlungen **(Koloskopie ≤10 Jahre oder FOBT ≤2 Jahre) in Ihrer Konsultation.**

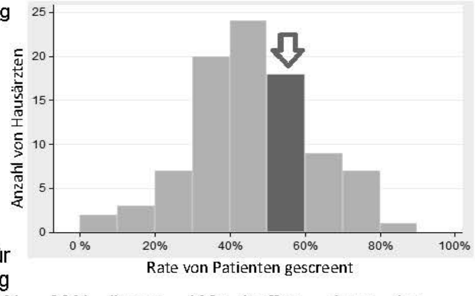

- **Ihre Ergebnisse**: 56% Ihrer Patientenwurden wurden für Dickdarmkrebs gescreent.
- **Implikationen**: Das Ziel unserer Studie ist, dass langfristig 100% der in Frage kommenden Patienten entscheiden können, ob sie ein Screening wollen oder nicht (einschließlich Ablehnung, Screening mit Koloskopie oder FIT, Verschiebung der Entscheidung). Wenn man bedenkt dass <5% eine Kontraindikation für ein Screening haben und 10-30% lehnen ein Screening ab, sollte die ideale Screeningrate bei etwa 70% -80% liegen. Wir hoffen, dass das Interventionsmaterial hilfreich sein kann, um Ihr Ziel zu erreichen.

Abbildung 2 (A et B) :

A - Anteil der Patienten bei denen **in der Vergangenheit ein FOBT durchgeführt wurde** im Vergleich zur Koloskopie (FOBT / Koloskopie).

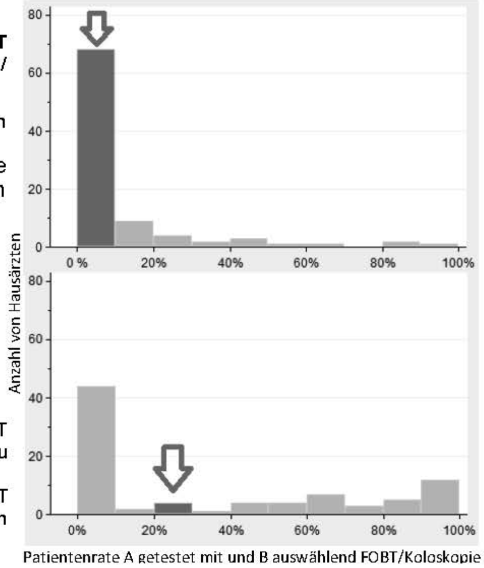

- **Ihre Ergebnisse**: Von all Ihren gescreenten Patienten, wurden keine (0%) mit einem FOBT getestet
- **Implikationen:** Diese Rate spiegelt wahrscheinlich die Überlegenheit der Kolonoskopie im Vergleich zu dem alten Guajak-FOBT-Test wider (z. B. Hemoccult). Der immunologische FIT-Test ist jetzt eine Alternative zur Koloskopie. Teil B zeigt wie diese Raten in Zukunft bei Ihren Patienten sich ändern können.

B - Prozentsatz der Patienten, **denen FOBT verordnet wurde**, im Vergleich zur Koloskopie (FOBT / Koloskopie) **nach Gespräch** über Darmkrebs-Screenings.

- **Ihre Ergebnisse:** Nach dem Gespräch haben Sie ein FOBT bei 27% Ihrer Patienten die sich entschieden haben getestet zu werden verschrieben.
- **Implikationen:** Ihre Offenheit für die Verschreibung der FOBT ist willkommen. Nach der Verteilung der beigefügten Entscheidungshilfe hat eine in der Schweiz durchgeführte Studie gezeigt, dass die Hälfte der Patienten den FOBT und die andere Hälfte die Koloskopie gewählt hat. Sie können die Entscheidungshilfe mit ihren Patienten verwenden um zu sehen, ob diese Proportionen bei Ihrem Patienten in der nächsten Datensammlung in diesem Herbst bestätigt werden.

Figure 3 – Prozentsatz der Patienten, die nach einem Gespräch über Darmkrebs-Screening eine Untersuchung abgelehnt haben.

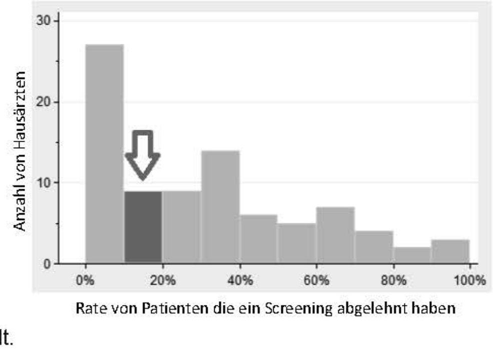

- **Ihre Ergebnisse:** 16% der Patienten mit denen Sie das Screening besprochen haben, weigerten sich getestet zu werden
- **Implikationen :** Ihre Ablehnungsraten liegen unter dem Durchschnitt Ihrer Sentinella-Kollegen. Unsere Analysen von Sentinella-Daten legen nahe, dass Ärzte die beide Methoden verschreiben, eine niedrigere Ablehnungsrate haben als diejenigen die nur eine Koloskopie verschreiben. Es ist möglich, dass Ihre Offenheit für die Verschreibung des FOBT sich in dieser geringeren Ablehnungsrate widerspiegelt.
