# Supplementary material for: Promoting shared decision-making in colorectal cancer screening in primary care: A cluster randomized controlled trial
Source: PLoS One. 2026 Jun 9;21(6):e0351069. doi: 10.1371/journal.pone.0351069 (PMC13249137; doi:10.1371/journal.pone.0351069)

S6 Fig. Modified primary outcome: Proportion of PCP with at least one patient tested with FOBT before discussion (without considering FOBT prescribed during the data collection period)

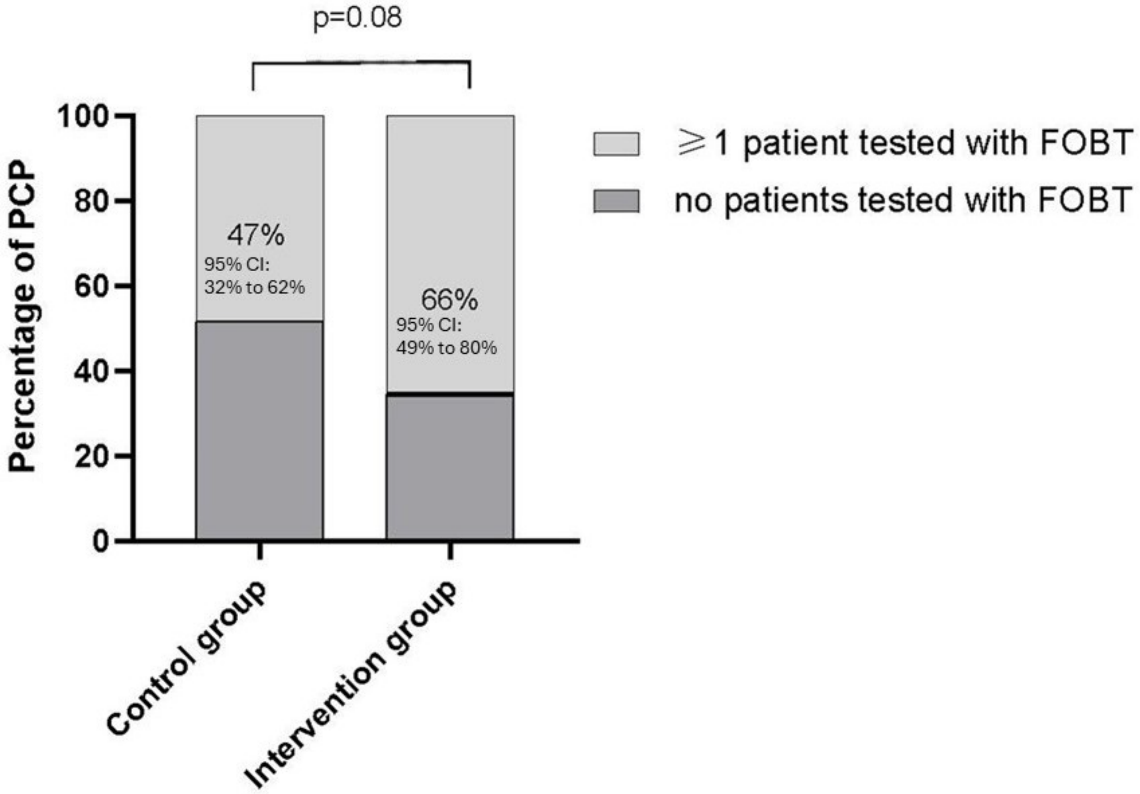

Supplement: S6 Fig — (PDF) [file pone.0351069.s011.pdf]
